# Subjective ulnar nerve dysfunction commonly following open reduction, internal fixation (ORIF) of distal humeral fractures and in situ decompression of the ulnar nerve

**DOI:** 10.1007/s11751-016-0271-5

**Published:** 2016-12-01

**Authors:** Birgitta Svernlöv, Jens Nestorson, Lars Adolfsson

**Affiliations:** 1 Department of Plastic Surgery, Hand Surgery and Burns, Linköping University Hospital, 581 85 Linköping, Sweden; 2 Faculty of Experimental and Clinical Medicine, Linköping University, Linköping, Sweden; 3 Department of Orthopedic Surgery, Linköping University Hospital, 581 85 Linköping, Sweden; 4 Faculty of Health Science, Linköping University, Linköping, Sweden

**Keywords:** Fracture, ORIF, Transposition, Humeral, Dellon, McGowan

## Abstract

The aim of this retrospective study was to investigate the frequency of persistent ulnar affection in patients who underwent open reduction and internal fixation (ORIF) of distal humeral fractures without ulnar nerve transposition or mobilisation. Eighty-two patients (53 women), mean age 62 years, were, at a mean of 48 months, reviewed through medical records and a subjective evaluation form concerning ulnar nerve problems. Ulnar nerve affliction, in most cases regarded as mild, was experienced by 22 patients (27%; 14 women) and significantly associated with multiple surgeries. Three patients had been operated with late neurolysis and one with transposition without reported improvement. The proportion of ulnar nerve dysfunction was equally common regardless of medial or lateral plating. ORIF with plate fixation and without ulnar nerve transposition seems to be an acceptable option for patients with distal humeral fractures. The frequency of ulnar nerve affection in our series does not appear higher than previously reported. Subjective ulnar nerve symptoms were, however, relatively common and appear related to the trauma itself, the surgery, or the post-operative management which highlights the need for further analysis of these factors.

## Introduction

Fractures of the distal humerus in adults are estimated to represent 2% of all fractures and are thus relatively infrequent [1]. Usually operative treatment with accurate anatomic reduction and stable internal fixation is indicated [2–6]. Dual-plate fixation has become the treatment of choice for most surgeons during which procedure the ulnar nerve has to be mobilised to some extent and ulnar nerve dysfunction is a common complication following surgical treatment of distal humeral fractures [7]. The ulnar nerve is at high risk of being injured at the initial trauma during surgery and may also be affected by post-operative scar formation [8–12]. The reported incidence of post-operative ulnar neuropathy varies between 0 and 51% with an average of 13% [3, 7, 13–19]. Many authors advocate routine anterior transposition of the nerve [7, 13, 16, 18, 20–24], but some support the idea of placing the nerve back into its epicondylar groove after the internal fixation is completed [3, 14, 19].

The aim of this study was to investigate the frequency of persistent ulnar affection in patients who underwent open reduction and internal fixation (ORIF) of distal humeral fractures without ulnar nerve transposition or mobilisation.

## Materials and methods

Between January 2003 and June 2013, 161 patients with distal humeral fractures were operated at our centre. Out of these, 116 adults were treated with internal plate fixation using bilateral or unilateral plates and screws. There were 35 men and 81 women with an average age of 63 years (SD 18.31, range 21–93 years).

The patients’ medical records, comprising information about demographics, operative and hospital information, and any complication that occurred in the immediate or later post-operative period, were reviewed. Twenty-four patients, four males and twenty females, were deceased, and two patients, one man and one woman, could not be reached due to foreign citizenships. The rest of the patients were contacted by phone and offered a follow-up; four women and one man were unable to cooperate due to vascular dementia or mental handicap of other origin. Another three women could not participate due to language difficulties. This left 82 eligible patients (71%), 29 males and 53 females, available for follow-up. The average age was 63 years (SD 16.40, range 21–89 years). The mean age of the men was 62 years (SD 18.31, range 18–89 years), and that of the women was 63 years (SD 16.48, range 21–89 years). The average follow-up was 49 months (SD 27.85, range 14–118 months), 48 months (SD 31.41, range 14–118 months) for the male patients, and 49 months (SD 26.21, range 17–115 months) for the female patients.

The mechanism of injury included 49 simple falls, 11 bicycle accidents, ten falls from a height, six motor vehicle accidents, four rotation accidents (e.g. arm wrestling), one skiing accident, and one with a direct blow from a girder. Four were open fractures, and one patient had ipsilateral forearm fractures. The ulnar nerve function was, according to the medical records, preoperatively intact in all cases.

The preoperative images, including CT scans, were examined, and the fractures classified in accordance with the Arbeitsgemeinschaft für Osteosynthesefragen (AO) system (Table 1) [25].

**Table 1:** Fracture types according to the *Arbeitsgemeinschaft für Osteosynthesefragen* (*AO*) *classification* [25] and demographics

AO classification	Gender	Mean age	Ulnar nerve affection (*n* = 22)	
	M/F		M/F	M/F (%)
*A* (*n* *=* *11*)				
A2:1 1	0/1	32	0/0	0
A2:3 2	0/2	83	0/0	0
A3:1 1	0/1	69	0/0	0
A3:2 4	2/2	56	1/1	4.5/4.5
A3:3 3	0/3	62	0/1	0/4.5
*B* (*n* = *13*)				
B1:3 5	1/4	63	0/1	0/4.5
B2:1 1	1/0	60	0/0	0
B2:3 3	1/2	68	0/0	0
B3:1 1	0/1	66	0/0	0
B3:3 3	2/1	67	2/1	9.0/4.5
*C* (*n* *=* *58*)				
C1:2 7	2/5	65	0/2	0/9.0
C1:3 1	0/1	85	0/0	0
C2:1 4	2/2	67	0/1	0/4.5
C2:2 16	5/11	64	1/4	4.5/18.2
C2:3 14	6/8	61	2/1	9.0/4.5
C3:1 1	0/1	54	0/0	0
C3:2 11	4/7	61	1/2	4.5/9.0
C3:3 4	2/2	59	0/1	0/4.5

*N* = 82

Seventy patients had been operated with bilateral plating using the parallel concept with plates on each column at an angle of approximately 160° between the plates. In two patients the fracture was stabilised with a solitary medial plate, and in ten patients a lateral plate was the only implant used. The exposure included a mid-line triceps split in 45 patients, an olecranon osteotomy in 19, and a lateral or antero-lateral approach in 15 patients. Three patients had a concomitant fracture of the olecranon that was used for approach to the fracture.

The ulnar nerve was identified and decompressed (in situ) from the arcade of Struthers to the medial ulno-humeral joint line. When a medial plate was used, the ulnar nerve was carefully elevated from the humeral metaphysis and the medial intermuscular septum allowing for the plate to be slid underneath. The nerve was elevated together with a sleeve of perineural soft tissues. In all cases operated through a lateral or antero-lateral approach, the triceps and the ulnar nerve had been left undisturbed.

The patients were immobilised in a posterior plaster splint during two to three days. Active exercises monitored by a physiotherapist were initiated immediately after removal of the plaster. Light activities of daily living were allowed at all times, while load bearing and strengthening exercises begun after 6 weeks.

**Fig. 1:**
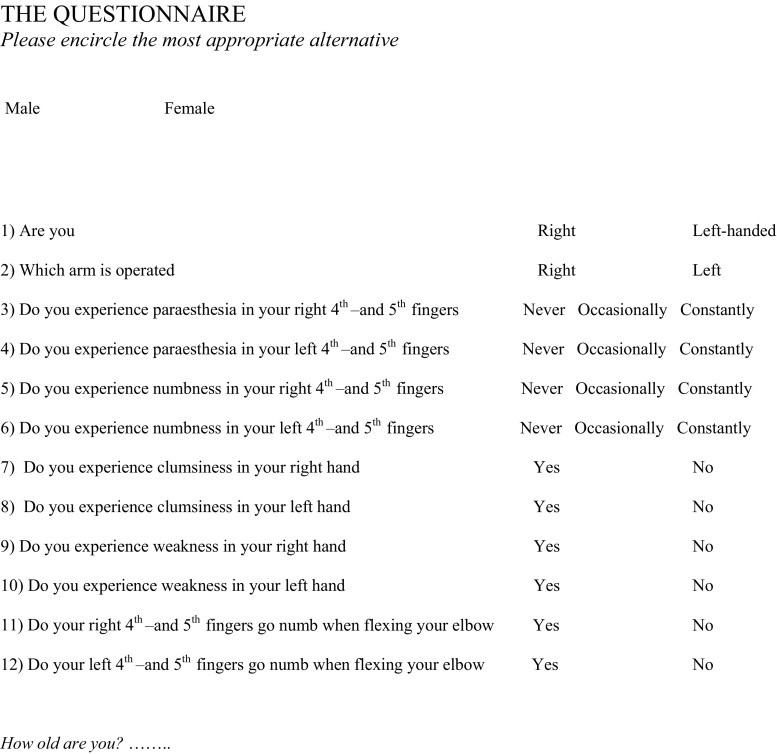
The subjective patient rated questionnaire concerning possible ulnar nerve affliction based on the systems by McGowan and Dellon [30, 31]

The follow-up consisted of a questionnaire (Fig. 1) addressing subjective symptoms from the ulnar nerve, such as occasional or constant numbness or paraesthesias in the 4th and 5th finger of the hand of the operated elbow; furthermore, experience of subjective weakness and clumsiness in the actual hand; and whether or not the fingers in question went numb in connection with elbow flexion. For each item the alternatives present or absent could be chosen. A diagnosis of ulnar nerve dysfunction was decided when intermittent paraesthesia and numbness in the 4th and 5th fingers, aggravated by elbow flexion, were reported and classified as a mild affection. Intermittent numbness and paraesthesia with additional weakness and clumsiness were graded as moderate and constant problems, including all these symptoms, as severe nerve affection.

The questionnaire was sent out by mail to the patients with a prepaid return envelope together with a cover letter and an informed consent form for signature to participate in the study. The participants were reassured of the confidential nature of the study.

An independent researcher (BS) carried out the study, which was approved by the regional committee for medical ethics (Dnr 2010/171-31).

Statistical analyses were made using STATISTICA v.12.0 StatSoft, Inc. The Chi square test was used to test the difference in proportions of ulnar nerve affliction between all patients and between those who had been subjected to re-operations; between the different fracture types; between men and women; between patients operated with or without the use of a medial plate; and between patients operated with or without an olecranon osteotomy. *T* tests for independent groups were used to test whether there was an age difference between patients presented with ulnar nerve affliction or not. *p* values <0.05 were considered statistically significant.

## Results

In reference to the AO classification, 11 fractures were type A, 13 type B, and the remaining 58 type C fractures (Table 1) [25].

In accordance with the medical histories, there were 16 patients with ulnar nerve affection diagnosed during the post-operative follow-up period. At the last follow-up, on average 4 years, 22 patients (27%; 3 AO type A, 4 type B, 15 type C), 14 women and eight men, reported symptoms based on the criteria for ulnar nerve dysfunction, according to the questionnaire. Thirteen patients suffered from mild affections with only occasional paraesthesia and numbness in the 4th and 5th fingers, particularly during elbow flexion; four presented with intermittent numbness or paraesthesias in the 4th and 5th fingers and additionally subjective weakness and/or clumsiness in the hand and were considered as moderately affected. Five patients reported constant problems with all these symptoms and were regarded as severely affected (Table 2).

**Table 2:** Ulnar nerve affection related to fracture type, gender (male/female), mean age, re-operation, bilateral or lateral plates, and olecranon osteotomy

Nerve affection (*n* = 22)	Fracture type			Mean age	Re-operation	Bilateral plates	Lateral plates	Olecranon osteotomy
	A (*n* = 3)	B (*n* = 4)	C (*n* = 15)	M/F (56/63)	M/F (*n* = 13)	M/F (*n* = 19)	M/F (*n* = 3)	M/F (*n* = 7)
Mild (*n* = 13)	2	2	9	54/63	4/4	4/7	1/1	1/3
Moderate (*n* = 4)	0	2	2	70/54	1/2	1/2	1/0	0/1
Severe (*n* = 5)	1	0	4	46/70	1/1	1/4	0/0	0/2

Numbers presented are patients

Four of these 22 patients had undergone a second procedure with a neurolysis, in one patient combined with subcutaneous transposition, in connection with hardware removal. No improvement was reported following these procedures. Ulnar nerve affliction was significantly associated with multiple surgeries (*p* < 0.01). No significant difference was found between gender (*p* > 0.93) or age (*p* > 0.54) and ulnar nerve problems (Table 2).

Post-operative ulnar nerve dysfunction was not related to fracture type (*p* = 0.50). Ten patients had been operated without the use of a medial plate in which cases the fracture had been stabilised using lateral implants only. There was no significant difference in ulnar nerve symptoms between patients operated with bilateral plates or a single ulnar plate on the medial column (*p* < 0.81) and those who were treated with only a lateral plate nor was there any significant difference in ulnar nerve problems between those operated with an olecranon osteotomy or not (*p* < 0.54; Table 2).

Eight patients presented with radial palsy in connection with the injury. None of these were surgically explored or repaired, and all subsequently resolved without residual symptoms. Twenty-five patients (30%) had undergone reoperation; 15 with hardware removal, four of which with concomitant ulnar neurolysis due to nerve symptoms. Three patients had been operated with bone graft and new osteosynthesis, two due to non-union of the distal humeral fracture, and one because of non-union of an olecranon osteotomy. All of them subsequently went on to union. Two patients were operated with wound revision due to deep infection. Two patients developed avascular bone necrosis of the distal humerus, one was treated with resection of capitellar fragments, and one, with affection of the entire joint surface, was treated with a hemiprosthesis. Three patients were operated with resection of heterotopic bone formation interfering with mobility.

## Discussion

Ulnar neuropathy at the elbow is the second most frequent focal peripheral neuropathy of the upper limb, usually presenting with tingling and paraesthesia in the little and ring fingers and weakened hand grip. In the present study, 27% of the patients operated with internal fixation of a distal humeral fracture reported symptoms from the ulnar nerve at a 4-year follow-up.

The issue of ulnar nerve affection associated with internal fixation of distal humeral fractures has been addressed in several studies resulting in varying conclusions; Shin and Ring [18] found a 22% rate of post-operative ulnar nerve palsies after nerve transposition, and according to them, despite adequate release and transposition, irritation and transient sensory changes have occurred in up to 50% of patients in some series. In fact, after ulnar nerve transposition had been performed, 51% of transient ulnar neuropraxia was reported by Holdsworth and Mossad [14]. Furthermore, McKee et al. [5] calculated a 20% rate of ulnar neuropathy after the same procedure. Athwal et al. [26] concluded that 13% of post-operatively developed ulnar neuropathy might have been the result of the routine transposition of the ulnar nerve that they performed in connection with ORIF.

Chen et al. [27], comparing the incidence of ulnar neuritis with and without nerve transposition, recognised almost four times (33%) the incidence in those who underwent transposition. The authors concluded that transposition of the ulnar nerve may not be helpful in preventing the development of ulnar neuritis after distal humeral fractures, instead may place the patient at greater risk for neuritis. Kundel et al. [3], on the other side, after performing in situ release of the nerve in question described a 27% prevalence of ulnar neuropathy.

Worden and Ilyas [28], in a review study, identified a 38% incidence of late ulnar neuropathy in connection with ORIF and found no difference between in situ release and anterior transposition. In an attempt to detect factors associated with ulnar neuropathy, Wiggers et al. [19] diagnosed a 16% ulnar neuropathy, regardless of whether the nerve was transposed or not. Furthermore, Vazquez et al. [12] retrospectively evaluated distal humeral fractures treated with or without ulnar nerve transposition. They discovered, irrespective of procedure, the incidence of post-operative neuropathy to be 16% and concluded that transposition of the nerve did not significantly decrease the development of iatrogenic ulnar neuropathy. Additionally, Ruan et al. [11] randomly allocated patients to either anterior subfascial transposition or in situ decompression, of the ulnar nerve in conjunction with ORIF. They found that there was no significant difference between the groups.

The optimal handling of the ulnar nerve is unclear, but anterior transposition may not be necessary as part of the acute surgical treatment of displaced distal humeral fractures [12].

However, the true prevalence of ulnar nerve dysfunction after elbow injury is unknown, since authors of published studies have not successfully distinguished acute injury-related, acute surgery-related, and delayed (subacute or chronic) ulnar neuropathies, and furthermore, in most of these retrospective case series, careful evaluation of ulnar nerve function has not been included [18].

Wiggers et al. [19] looked for risk factors for post-operative ulnar neuropathy, including age, sex, implant over or below the medial epicondyle, and the total number of surgeries. They learned that columnar fracture and application of a medial plate were the only potential risk factor for iatrogenic post-operative ulnar neuropathy, but Vazquez et al. [12] were not able to identify any single factor that significantly contributed to ulnar neuropathy. They investigated transposition of the ulnar nerve or not, age, gender, presence of multiple procedures, use of olecranon osteotomy, poly-trauma, and open versus closed injury. We found no significant difference in ulnar nerve symptoms between patients in whom an ulnar plate was used and those treated with lateral implants only. This might indicate that the main cause is the trauma itself or that ulnar nerve symptoms could occur secondarily to post-operative immobilization, swelling, scarring, and thickening in the fibro-osseous tunnel. The only variable we detected associated with ulnar nerve affection was re-operation, but this association is hampered by the fact that the reason for reoperation in four cases was because of ulnar nerve symptoms.

The reoperation rate of 30% in our series corresponds with the literature, the results of which fall between 21 and 73% with the majorities in or around 40% [4, 7, 16, 17, 23, 27–29].

This study is impaired by some limitations that are related primarily to the inherent weakness of a retrospective report. There is no direct comparison with a group of patients randomly allocated to another treatment of the ulnar nerve in connection with the surgery. Differences in fracture types and trauma mechanisms may have had an impact on the susceptibility of ulnar nerve affection, but the material is of insufficient size for subgroup analysis. Another potential weakness is the subjective patient-rated questionnaire concerning possible ulnar nerve affliction, since this precludes any objective measures of dysfunction. On the other hand, the majority of complaints include minor sensory disturbance and discomfort that may not have been possible to appreciate by a clinical or neurophysiologic examination.

We decided to construct the questionnaire including all subjectively experienced factors previously described associated with an ulnar neuropathy at the level of the elbow, in reference to the examination systems proposed by McGowan [30] and Dellon [31]. A report of tingling, paraesthesia, numbness, clumsiness, weakness as well as increased symptoms associated with elbow flexion was regarded as definitive attributes of a nerve dysfunction. We are aware that comparison with other studies is difficult since many different methods for assessing ulnar nerve dysfunction have been used but since the main complaint of our patients was subjective sensation of intermittent sensory disturbance which is not objectively measurable, we believe that the method used is appropriate. The questionnaire is not validated in relation to other methods, and the results should therefore be cautiously interpreted.

The strengths of our study are the sample size of 82 patients, that all eligible patients participated, the surgeries were performed by experienced orthopaedists in a single centre, the follow-up period of 4 years appears reasonable, and that an independent reviewer, not involved in the surgeries, conducted the survey.

Future studies that objectively and reliably diagnose injury-related, surgery-related, and delayed (sub-acute or chronic) ulnar neuropathies or prospective randomized trials, using transposition or in situ release of the ulnar nerve with strict definitions and objective measures, would be valuable.

## Conclusion

ORIF without ulnar nerve transposition seems to be an acceptable option for patients with distal humeral fractures. Late ulnar nerve dysfunction was found to be a relatively common problem following surgically treated distal humeral fractures. The frequency of the discomfort in our study was somewhat disappointing but, according to what can be learnt from the literature, we do not believe that an anterior transposition of the nerve is preferable to in situ decompression.

## References

[1] Robinson CM, Bucholz RW, Heckman JD, Court-Brown CM (2006). Fractures of the distal humerus. Rockwood’s and Green’s fractures in adults.

[2] Jupiter JB, Neff U, Holzach P (1985). Intercondylar fractures of the humerus: an operative approach. J Bone Joint Surg Am.

[3] Kundel K, Braun W, Wieberneit J (1996). Intraarticular distal humerus fractures: factors affecting functional outcome. Clin Orthop Relat Res.

[4] Korner J, Lill H, Muller LP (2005). Distal humerus fractures in elderly patients: results after open reduction and internal fixation. Osteoporos Int.

[5] McKee MD, Veillette CJ, Hall JA (2009). A multicenter, prospective, randomized, controlled trial of open reduction -internal fixation versus total elbow arthroplasty for displaced intra-articular distal humeral fractures in elderly patients. J Shoulder Elbow Surg.

[6] Adolfsson L, Nestorson J (2012). The Kudo humeral component as primary hemiarthroplasty in distal humeral fractures. J Shoulder Elbow Surg.

[7] Gofton WT, MacDermid JC, Patterson SD (2003). Functional outcome of AO type C distal humeral fractures. J Hand Surg Am.

[8] Webb LX, Bucholz RW, Heckman JD, Court-Brown CM (2001). Fractures of the distal humerus. Rockwood’s and Green’s fractures in adults.

[9] Huang TL, Chiu FY, Chuang TY (2005). The results of open reduction and internal fixation in elderly patients with severe fractures of the distal humerus: a critical analysis of the results. J Trauma.

[10] McCarty LP, Ring D, Jupiter JB (2005). Management of distal humerus fractures. Am J Orthop.

[11] Ruan HJ, Liu JJ, Fan CY (2009). Incidence, management, and prognosis of early ulnar nerve dysfunction in type C fractures of distal humerus. J Trauma.

[12] Vazquez O, Rutgers M, Ring DC (2010). Fate of the ulnar nerve after operative fixation of distal humerus fractures. J Orthop Trauma.

[13] Wang KC, Shih HN, Hsu KY (1994). Intercondylar fractures of the distal humerus: routine anterior subcutaneous transposition of the ulnar nerve in a posterior operative approach. J Trauma.

[14] Holdsworth BJ, Mossad MM (1990). Fractures of the adult distal humerus. Elbow function after internal fixation. J Bone Joint Surg Br.

[15] Södergard J, Sandelin J, Böstman O (1992). Postoperative complications of distal humeral fractures. 27/96 adults followed up for 6 (2–10) years. Acta Orthop Scand.

[16] Gupta R, Khanchandani P (2002). Intercondylar fractures if the distal humerus in adults: a critical analysis of 55 cases. Injury.

[17] Soon JL, Chan BK, Low CO (2004). Surgical fixation of intra-articular fractures of the distal humerus in adults. Injury.

[18] Shin R, Ring D (2007). The ulnar nerve in elbow trauma. J Bone Joint Surg Am.

[19] Wiggers JK, Brouwer KM, Helmerhorst GT (2012). Predictors of diagnosis of ulnar neuropathy after surgically treated distal humerus fractures. J Hand Surg Am.

[20] Jupiter JB, Barnes KA, Goodman LJ (1993). Multiplane fracture of the distal humerus. J Orthop Trauma.

[21] Jupiter JB (1995). Complex fractures of the distal part of the humerus and associated complications. Instr Course Lect.

[22] Ring D, Jupiter JB (1999). Complex fractures of the distal humerus and their complications. J Shoulder Elbow Surg.

[23] Ring D, Gulotta L, Jupiter JB (2003). Unstable non-union’s of the distal part of the humerus. J Bone Joint Surg Am.

[24] Ilyas AM, Jupiter JB (2008). Treatment of distal humerus fractures. Acta Chir Orthop Traumatol Cechoslov.

[25] Müller ME, Nazarian S, Koch P (1990). Humerus = 1. The comprehensive classification of fractures of long bones.

[26] Athwal GS, Hoxie SC, Rispoli DM (2009). Precontoured parallel plate fixation of AO/OTA type C distal humerus fractures. J Orthop Trauma.

[27] Chen G, Liao Q, Luo W (2011). Triceps-sparing versus olecranon osteotomy for ORIF: analysis of 67 cases of intercondylar fractures of the distal humerus. Injury.

[28] Worden A, Ilyas AM (2012). Ulnar neuropathy following distal humerus fracture fixation. Orthop Clin North Am.

[29] Huang JI, Paczas M, Hoyen HA, Huang JI, Vallier HA (2011). Functional outcome after open reduction internal fixation of intra-articular fractures of the distal humerus in the elderly. J Orthop Trauma.

[30] McGowan AJ (1950). The results of transposition of the ulnar nerve for traumatic ulnar neuritis. J Bone Joint Surg Br.

[31] Dellon AL (1989). Review of treatment results for ulnar nerve entrapment at the elbow. Hand Surg Am.

